# Designing High-Fidelity Mobile Health for Depression in Indonesian Adolescents Using Design Science Research: Mixed Method Approaches

**DOI:** 10.2196/48913

**Published:** 2023-07-03

**Authors:** Mila Shania, Putu Wuri Handayani, Sali Asih

**Affiliations:** 1 Faculty of Psychology University of Indonesia Depok Indonesia

**Keywords:** mobile health, mental health, user interface, design science research, Indonesia, digital app, mHealth, depression, pandemic, adolescents

## Abstract

**Background:**

COVID-19 mitigation protocols, enacted to control the pandemic, have also been shown to have a negative impact on mental health, including the mental health of adolescents. The threat of being infected by SARS-CoV-2 and substantial changes in lifestyle, including limited social interaction due to stay-at-home orders, led to loneliness as well as depressive symptoms. However, offline psychological assistance is restricted, as psychologists are bounded by mitigation protocols. Further, not all adolescents’ guardians are open to their children attending or have the means to pay for psychological service; thus, adolescents remain untreated. Having a mobile health (mHealth) app for mental health that uses monitoring, provides social networks, and delivers psychoeducation may provide a solution, especially in countries that have limited health facilities and mental health workers.

**Objective:**

This study aimed to design an mHealth app to help prevent and monitor depression in adolescents. The design of this mHealth app was carried out as a high-fidelity prototype.

**Methods:**

We used a design science research (DSR) methodology with 3 iterations and 8 golden rule guidelines. The first iteration used interviews, and the second and third iterations used mixed method approaches. The DSR stages include the following: (1) identify the problem; (2) define the solution; (3) define the solution objective; (4) develop, demonstrate, and evaluate the solution; and (5) communicate the solution. This study involved students and medical experts.

**Results:**

The first iteration resulted in a wireframe and prototype for the next iteration. The second iteration resulted in a System Usability Scale score of 67.27, indicating a good fit. In the third iteration, the system usefulness, information quality, interface quality, and overall values were 2.416, 2.341, 2.597, and 2.261, respectively, indicating a good design. Key features of this mHealth app include a mood tracker, community, activity target, and meditation, and supporting features that complement the design include education articles and early detection features.

**Conclusions:**

Our findings provide guidance for health facilities and to design and implement future mHealth apps to help treat adolescent depression.

## Introduction

Mental disorders are growing global problems [1], and approximately 80% of the burden will be in low and middle-income countries [2]. The World Health Organization [3] predicted that depression was likely to be the second largest global illness burden in 2020 and may become the top global illness burden by 2030. The consequences of depression are more pronounced in adolescents (12-25 years) even if the depression has been previously treated [4].

According to the Indonesian Ministry of Health [5], mental disorders result in the highest number of years lost due to illness or disability. The Indonesian Ministry of Health [5] states that the prevalence of mental and emotional disorders in the young generation (between the ages of 15 years and 24 years) has steadily increased since 2013. Further, reports indicate that around 12 million Indonesians aged 15 years to 24 years experience depression. Suicide, one symptom of severe depression, has been reported for 0.8% and 0.6% of women and men, respectively, aged 15 years to 24 years (N=722,329) [5]. Mental disorders such as depression in adolescents also confer a cross-generational risk, affecting the health of depressed adolescents’ offspring when they become parents [6]. Although the number of adolescents experiencing depression continues to increase and the consequences of depression are long-lasting and can be fatal, many people, particularly the older generation, still do not consider this disorder important [7]. This may be related to a lack of information and understanding of the challenges experienced by adolescents. In addition, discrimination and stigma exacerbate people's negative views of someone who has depression [7]. Discrimination and stigma may be related to minimal literacy levels regarding mental health [7]. Families and relatives of people with psychiatric disorders tend to restrict the interaction of these people with people outside their families and even sometimes put them in an isolated room. Further, the number of psychological services in health facilities is limited. Based on a survey conducted by the Indonesian Clinical Psychology Association, approximately 1300 clinical psychologists offer services in health facilities for a nation with more than 200 million people [8]. The low number of mental health practitioners has resulted in limited health service access for preventing and managing depression in the community [3].

Mobile health (mHealth) apps may provide a solution for the limited availability of health services [9,10]. Moreover, Carter et al [11] stated that some digital interventions for depression showed improvements in depressive symptoms, quality of life, treatment adherence, and recovery. Furthermore, Graham et al [12] and Moberg et al [13] found that an mHealth app was effective for depression among patients. mHealth apps can assist with monitoring programs and provide easy access to data collection and storage [14]. Furthermore, to overcome the limited number of mental health workers, mental health apps can also use chatbots or artificial intelligence technology to provide more generalized support [15-17]. In 2015, the Indonesian Ministry of Health launched a mental health mHealh app; in this app, users can search for information related to the location of health facilities, search for general information, or submit a report. Users can also perform tests for the early detection of mental ill-health. However, the activities that users can do are still limited to the prevention stage, because it only presents general information [18]. The app is therefore inadequate for prevention and monitoring efforts. Our research aimed to overcome the existing deficiencies and may help reduce the level of depression in adolescents.

Previous studies have investigated mHealth apps specifically for depression, so our findings provide a reference for features, elements, and other information relevant to shaping depression-focused health apps, especially within an Indonesian context. According to Grist et al [19], only 6 apps for children and adolescents were available to download, and none had undergone any research evaluation. Among previous studies, Kenny et al [20] targeted adolescents, van Dooren et al [21] applied gamification and a persuasive game design model, and Narváez et al [22] applied a user-centered design methodology. Merry et al [17] developed a digital ecosystem using a chatbot that can be integrated into school and health care systems, and they found that the development of an integrated and equitable digital system takes time and collaboration. Therefore, it is very important to design an app that is easy to use and has many benefits so that users want to continue using it [23,24]. Thus, our research question was how to design an mHealth app to manage depressive symptoms in adolescents. This research can be used as a basis for app developers to develop mental health apps, especially mental health apps that focus on depression in adolescents.

## Methods

### Study Design

This study used the design science research (DSR) paradigm methodology from Peffers et al [25] and 8 golden rule guidelines from Shneiderman and Plaisant [26] to design mHealth (Modi) apps that suit users’ needs, especially in Indonesia. Based on the work by Peffers et al [25], the DSR methodology has the main objective of creating and evaluating artifacts—or more specifically solutions—to solve and address problems facing organizations. This methodology is in accordance with rigorous scientific research methods and includes various techniques, principles, and procedures for designing and developing solutions to effectively solve the problems at hand [25]. Furthermore, the DSR is relevant to research on developing mHealth and supports creating innovative artifacts to solve real-world problems [27]. Shneiderman and Plaisant [26] created rules or principles to design an app interface. Shneiderman and Plaisant [26] formulated the principle of 8 golden rules, namely (1) strive for consistency, (2) enable frequent users to use shortcuts, (3) offer informative feedback, (4) design dialogue to yield closure, (5) offer simple error handling, (6) permit easy reversal of actions, (7) enable an internal support locus of control, and (8) reduce short-term memory load.

Based on DSR, the methodology was applied in 3 iterations. The first iteration was a low-fidelity prototype or wireframe design to test the suitability of the solution for addressing the problem and user needs. The first iteration results from the interview evaluation method were used as input for the second iteration, which used the System Usability Scale (SUS) model from Nielsen [28] to measure the design’s usability. In addition, we used the SUS across iterations to measure design improvements. The SUS is one of the most widely used frameworks and has been translated into several languages, including Indonesian [29,30]. The third iteration used the Post-Study System Usability Questionnaire (PSSUQ) model to measure the level of system usefulness, information quality, and interface quality. The PSSUQ framework is widely known and recommended for measuring usability in mobile-based apps [31,32].

We used both quantitative and qualitative methods for this study. We used a qualitative approach to enrich and improve the validation of the design results from each iteration so that the designs could be in accordance with the needs of promotive, preventive, and monitoring efforts for depression in adolescents. User-centered design can increase user engagement when using the app [33]. We first formulated the problem and conducted a literature review, then we conducted the DSR methodology stages, each of which had 3 iterations. The DSR methodology’s 5 stages are as follows: (1) identify the problem; (2) define the solution; (3) define the solution objective; (4) develop, demonstrate, and evaluate the solution; and (5) communicate the solution [25]. All respondents involved in this study have used the mHealth app and are technology savvy.

We identified the problem through a literature review; by conducting interviews with potential users, which, in this study, comprised 9 people who had experienced depression; and by examining several mental health apps that focused on teleconsulting services, meditation, and physical activity monitoring (purposive sampling). When designing an app, user experience (UX) factors, such as social interaction and accessibility, are also included. Social interaction and accessibility are some aspects of UX that are considered by users in mental health mobile technologies [20]. The opportunity to interact socially to share problems and give each other advice is a point that needs to be considered in designing a mental health app. Using mental health apps should be easily accessible and easy to use. We also link between the results of participant interviews and health app observations.

The next stage involved demonstration. First, we developed a questionnaire to test the usability framework. Next, we conducted a design demonstration accompanied by readability tests conducted with 5 patient respondents from different backgrounds, to ensure that the questionnaire distributed at the next stage would be understood by all patient groups. Patient respondents were adolescents aged 12 years to 25 years who had experienced stress or depression. The demonstrations and readability testing also involved medical experts, including doctors, psychiatrists, psychologists, nurses, and lecturers. The first iteration involved interviews with 2 medical experts and 2 patients or users with a history of depression. In the second iteration, we conducted interviews with 1 expert and 3 patients. In the third iteration, we conducted the demonstration with 1 expert and 3 patients. The results of the readability test produced correct writing rules and use of language that is easy to understand without changing the meaning of the question.

In the evaluation and communication stages, we conducted second interviews with respondents from the previous demonstration stage and administered questionnaires that had been revised based on respondent feedback from the readability test. The interviews were used to dig deeper into participants’ responses to the app design. Quantitative data were obtained from the questionnaires, which began with an explanation and exploration of the app design according to the task or scenario that was the focus (onboarding, mood tracking, activity targets, meditation, or early detection). Next, the questionnaire items asked about the framework used, which was the SUS framework for the second iteration and the PSSUQ framework for the third iteration. The communication stage was conducted in parallel with the evaluation stage and conveyed the results obtained in 1 iteration to the relevant patient and professional stakeholders.

### Analysis Methods

For the medical professional and user interview data, we used content analysis to describe the requirements related to the mental health app. This analysis is usually appropriate when existing theory or research literature on a phenomenon is limited, and researchers immerse themselves in the data to allow new insights to emerge [34]. Thus, we grouped the needs of each idea and determined priorities for those most needed. The PSSUQ analyses used the average of the Likert scores, and the SUS analyses used the calculated SUS score, which ranged from 0 to 100. The SUS results were obtained using measurements made by Bangor et al [35] and used measurement ranges so that the results could be easily understood. The obtained results were used to determine the level of satisfaction with the design.

### Research Instruments

The questionnaire used for iterations 2 and 3 was divided into 2 parts: The first included demographic characteristics and health app use information. Demographic items included email or cell phone number, age, domicile, and occupation. Items on mental health app use covered experience with using mental health apps and included the app name, advantages, and disadvantages. The second part included items that covered the framework used to test usability and items on stress management. The SUS framework used a 5-point Likert scale (strongly disagree=1, strongly agree=5), and the PSSUQ used a 7-point Likert scale (strongly disagree=1, totally agree=7). The instruments are described in Multimedia Appendix 1 and Multimedia Appendix 2.

### Ethical Considerations

All respondents provided written informed consent to participate, and the data submitted were anonymous. The respondent data can only be used for the purposes of this research. This research received ethical approval from the Faculty of Computer Science at Universitas Indonesia on September 21, 2020.

## Results

### Identify Problem and Define Solution Objectives

Respondent demographic characteristics are shown in Table 1. The additional data collected through interviews included depression management services for adolescents in treatment in Indonesia, problems participants experienced with treating depression, and how participants dealt with depression. One participant received treatment through a psychologist or psychiatrist, and the rest had not consulted a medical expert.

**Table 1:** Demographic characteristics of the respondents involved in the “identify problem” and “define objectives of the solution” stages.

Respondent number	Gender	Profile	Occupation	Interview date
Respondent 1	Female	Patient (17-24 years old)	Student	June 12, 2020
Respondent 2	Female	Patient (17-24 years old)	Student	June 16, 2020
Respondent 3	Female	Patient (17-24 years old)	Student	June 20, 2020
Respondent 4	Male	Patient (17-24 years old)	Student	June 20, 2020
Respondent 5	Female	Patient (17-24 years old)	Student	June 22, 2020
Respondent 6	Male	Patient (17-24 years old)	Student	June 27, 2020
Respondent 7	Female	Patient (17-24 years old)	Student	June 29, 2020
Respondent 8	Female	Patient (17-24 years old)	Student	June 30, 2020
Respondent 9	Female	Patient (17-24 years old)	Student	August 1, 2020

Interviews revealed that participants who had never had a consultation usually used the early detection feature on several websites, such as psycom.net and depression.org.nz. Two respondents mentioned several shortcomings of early detection on websites. First, the websites were only available in English and sometimes used vocabulary that was difficult to understand. Second, the available information from the results was limited; to access full results, users were required to provide an email address or commit to a subscription:

I also tried to test advice from friends, using the website. There are results there, but to see further results, you need to subscribe to an email, making you lazy. Then there is only English, so if you don't understand English, it's a bit difficult.

Respondent 1

Interview participants had all used the app to help treat their depression; 9 mentioned that mental health apps such as Kalm, Wysa, and Daylio helped them monitor their mood changes and to simply make friends:

Usually, I use 2 applications directly, because to find friends, I use Wysa and to monitor daily activities it's better to use Daylio. [Respondent 7]

Although various apps were used, all respondents stated that no mental health app met all their needs; therefore, respondents downloaded 2 or more apps. The unsuitable form of the app also made users feel that searching for depression-related information—articles or tips—was easier through general search portals rather than through apps.

### Design and Development

**Figure 1:**
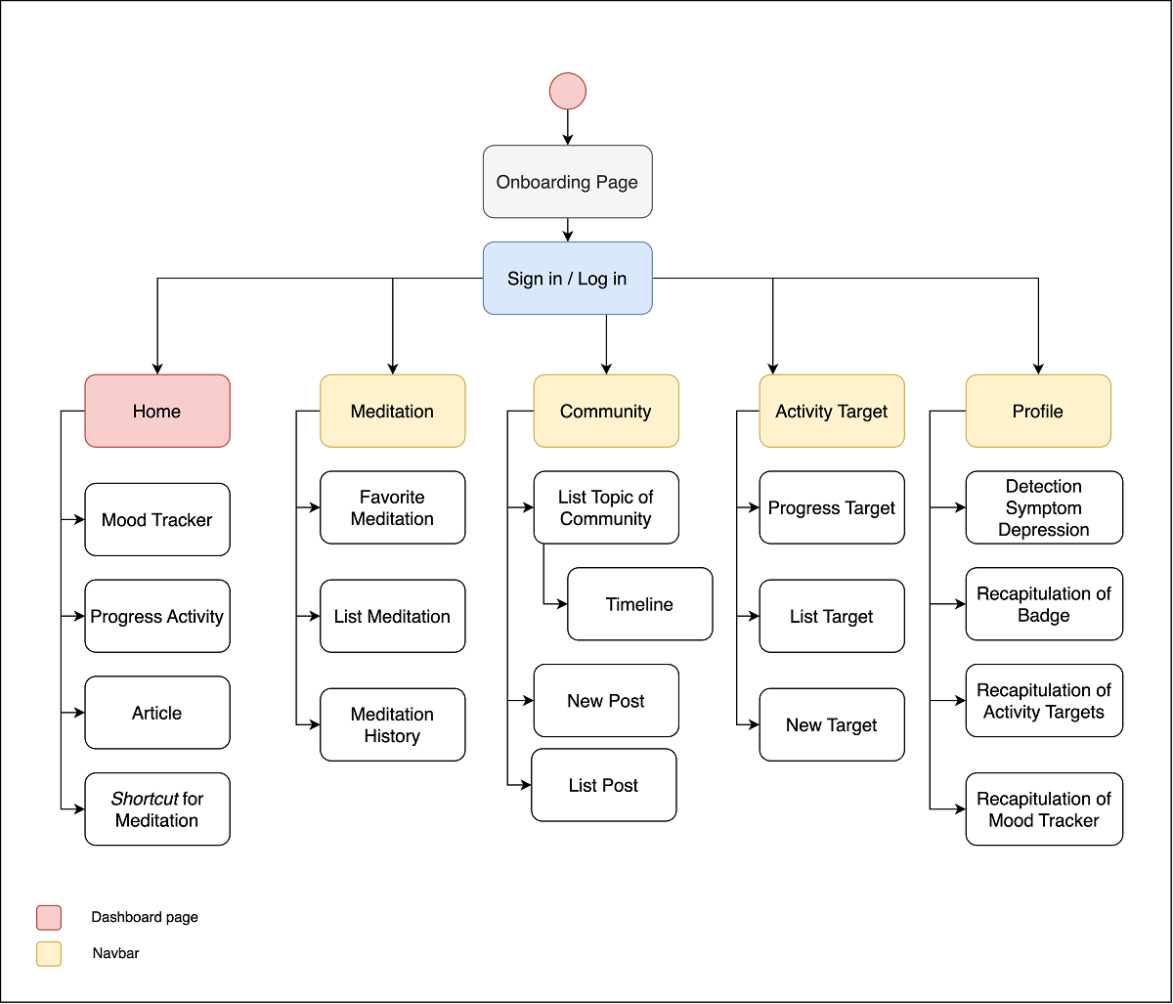
Information architecture.

The first iteration results were a low-fidelity prototype (wireframe), while the second and third iterations produced a high-fidelity prototype. The initial stage of the first iteration made observations about 12 mental health mHealth apps and concluded that several features are used by more than one app. We adopted the 6 most used features from those apps. The 4 features with the highest use frequency became the main features in this study’s app design (mood tracker, activity target, meditation, and community), and the remaining 2 featured (articles and early detection) were included as supporting features. The mood tracker is used to monitor the user's mood for a certain period. This feature is completed by the user himself or herself to assess the mood experienced by the user. The use of mood trackers helps with health management of people with mental disorders such as depression or bipolar disorder [36]. Mood trackers increase users' awareness and proactive self-regulation of their health care management through information about their health and the relationship between data and their condition [37-39]. The activity target feature is used to monitor activity targets created by users. Based on 5 applications that use the track activity feature, this feature helps users monitor the scheduling of their activity targets. The app provides a target template, such as sports, that can be selected. Doing activities such as exercise can help reduce symptoms of depression and has long-term beneficial effects [40,41]. The meditation feature serves to make it easier for users to perform meditation activities independently. In the benchmark app, the meditation feature provides several meditation templates based on topics that can be customized directly by the user. The meditation feature on the mobile app is effective at providing meditation that has an effect on reducing stress and increasing self-awareness and self-compassion [42]. The community feature is used for interactions between users. In the benchmark app, the community feature is formed to provide a place for users to communicate and support each other. The existence of social interaction is very important for maintaining physical and psychological health [43]. The article feature is to provide education to users regarding healing methods, tips, or information related to depression. In the benchmark app, the article feature displays information about depression in various forms such as tips or articles. Tips are given at the beginning of the app to increase awareness and enthusiasm when using the app. Motivational quotes are widely used in various media and therapy programs to promote positive thinking [44]. Early detection features can help users see the conditions they are experiencing. This feature can be an alternative early diagnosis for users who are still reluctant to go to health services or have difficulty getting access to health services, so that users can still get an overview of their mental health condition and immediately take preventive action so that the condition does not get worse. The implementation of the early detection feature used a patient health questionnaire in accordance with the guidelines for classification and diagnosis of mental disorders used in Indonesia [45,46]. Next, an information architecture was created to explain the features and flow of each app element (Figure 1).

### Demonstration

**Figure 2:**
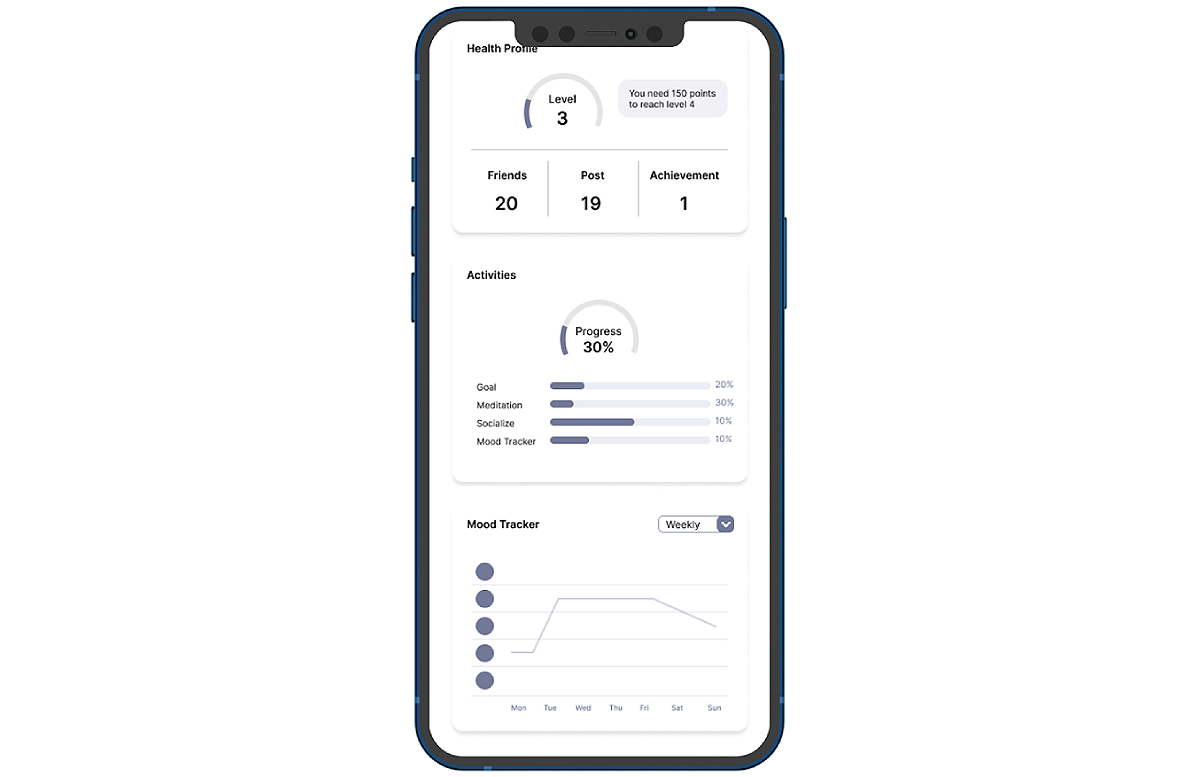
First iteration design.

**Figure 3:**
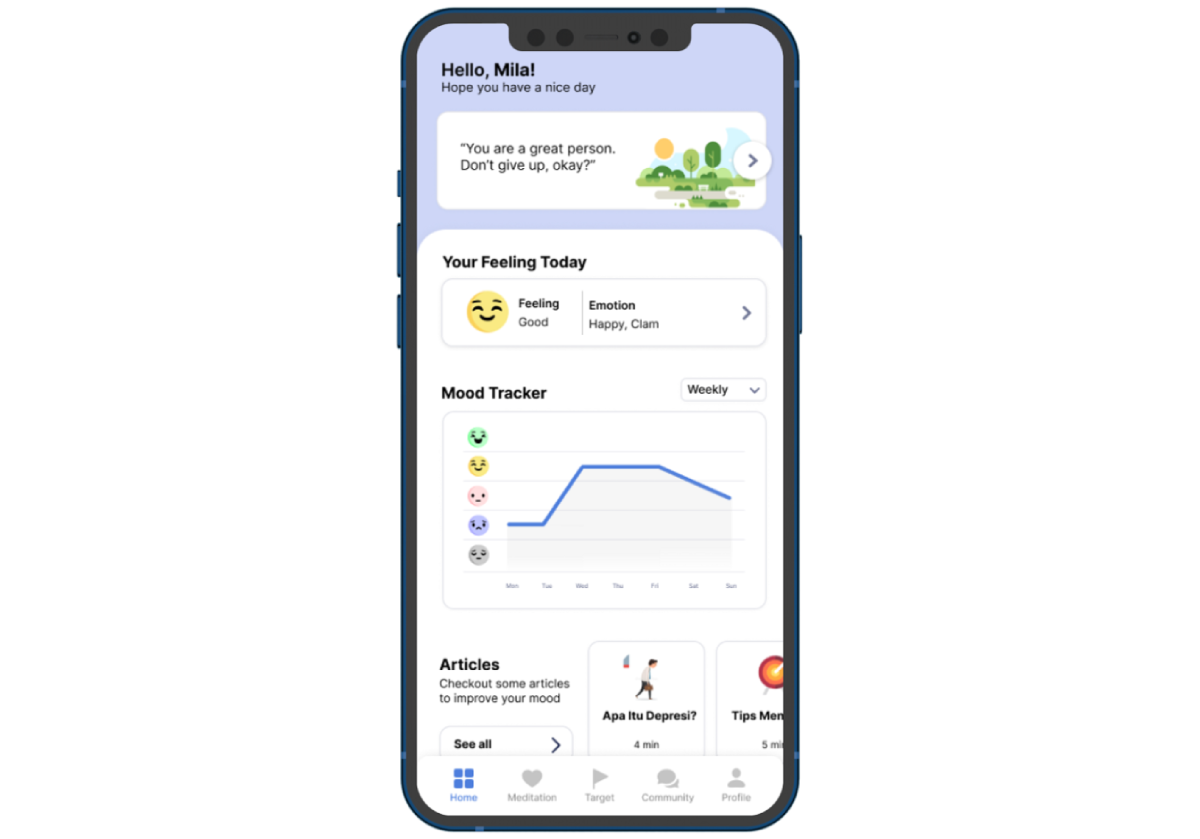
Second iteration design.

**Figure 4:**
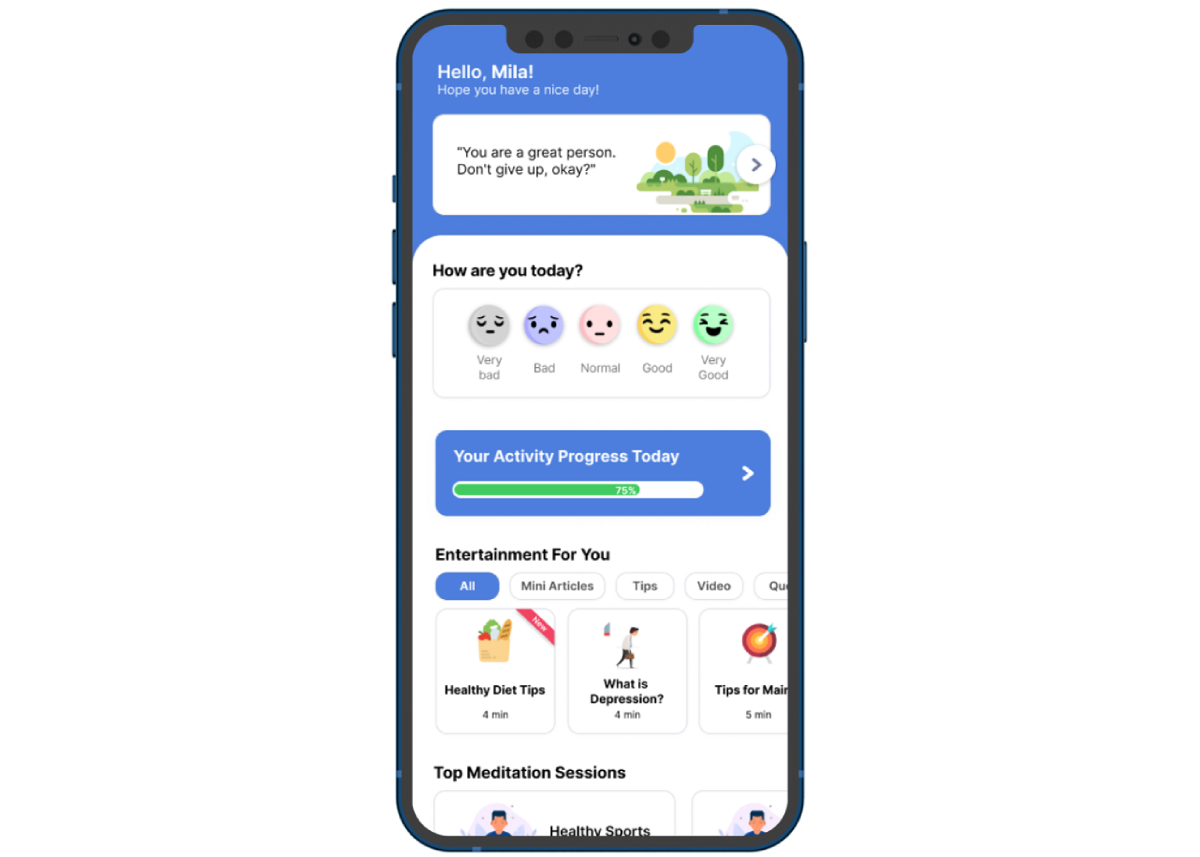
Third iteration design.

User interface design begins with designing the wireframe. Designing a low-fidelity prototype (wireframe) involves incorporating several features into the benchmark app. One example is the determination of the layout of the tips feature that is placed at the beginning of the app so that users can immediately see it when they open the app. The form of the low-fidelity prototype (wireframe) was then converted into a high-fidelity prototype (wireframe) in the second and third iterations. Figure 2 shows an example of a low-fidelity prototype (wireframe) from the first iteration and a high-fidelity prototype from the second (Figure 3) and third iterations of the mood tracker feature (Figure 4). Multimedia Appendix 3 presents the final main page design results with all features. Each design result could be seen and used by respondents, who evaluated the design through a questionnaire.

### Evaluation and Communication

The first iteration used an online video Zoom interview that was completed by 4 respondents. Respondent demographic characteristics are shown in Table 2. The 4 respondents provided feedback regarding the app’s main features (mood tracker, activity targets, community, and meditation) and showed agreement that these features can help treat depression in adolescents; however, there were suggestions for improvement of each feature.

**Table 2:** Demographic characteristics of the respondents interviewed in the first iteration.

Respondent number	Gender	Profile	Occupation	Interview date
Respondent 1	Female	Patient (17-24 years old)	Student	August 29, 2020
Respondent 2	Female	Patient (17-24 years old)	Student	August 30, 2020
Respondent 3	Female	Medical personnel (36-45 years old)	Psychologist	August 13, 2020
Respondent 4	Female	Medical personnel (17-24 years old)	Clinical psychology student	September 3, 2020

In the second iteration, we also used online interviews with 4 respondents via a video conference application to collect qualitative data. The interviews elicited suggestions that focused on the design. Implementation of main features such as a mood tracker, activity target, and community were deemed appropriate to users’ needs. However, 1 expert (Respondent 4) also emphasized that the meditation topic should possibly be reviewed:

Yes, it is useful to shorten the service time when he comes to the consult.

Respondent 4

Respondents also reacted favorably to the supporting features, articles, and early detection. The early detection feature provides them with an initial assessment of their mental health. Positive screening could indicate they have issues with their mental health even though they do not feel such issues. The screening could potentially provide them with options for self-healing or to seek further help from an expert. Interviewees believed that these 2 features helped educate patients and provided initial screening for their depressive conditions:

The early detection feature can help users perform initial screening of their condition. The results obtained can also be an assessment to see the level of depression symptoms that are felt.

Respondent 2

Early detection features are good. So, if you already feel certain symptoms, users can immediately try this feature and see if the depression level is high or not, so that they can be used as a reason for them to go to a psychologist or psychiatrist.

Respondent 3

Although the frequency of use is not as frequent as other features, the article feature helps those who need information related to depression, such as treatment, or tips for coping with depression.

Respondent 2

The design is good, but there are still pictures that don't match and the language is still not pleasant to read. Maybe it can be shortened, so that it is better to read.

Respondent 3

The 2 features were considered adequate in terms of flow but needed some improvement regarding the information presented. Respondents suggested that the provided information be processed again so that the language could be more easily understood by the user. They also suggested shortening the language, especially in the early detection feature, to maintain users’ focus and not increase their burden.

Moreover, online questionnaires provided quantitative data and were administered over 6 days, from October 10, 2020, to October 16, 2020. Demographic characteristics for the 108 respondents are shown in Table 3. The questionnaire data processing focused on the results of the usability testing framework, SUS, and feedback on the design. The SUS data assessment results indicated that the second iteration design was in the ”OK“ range (mean 67.27), suggesting that the design was good, although it still needed improvements in the element flow and layout to make the app easier to use.

In the third iteration, we also used online interviews with 4 respondents. Online questionnaires distributed over 8 days, from October 27, 2020, to November 3, 2020, provided quantitative data from 252 respondents. Respondent demographics are shown in Table 4.

The interviews provided design suggestions. The flow change from the previous design was considered more understandable. In addition, changes in appearance, especially layout, received positive reactions. The interviewees indicated that space given to each feature was decreased but still informative. The use of color was also considered improved over the previous design. More concise information was also easier to understand than in the previous design:

I said, the design is good. Features easy to understand.

Respondent 2

...I already understand the design better; the colors used are more pop-up than before, so it's easier to see. Statement is also simple but easy to understand.

Respondent 1

Respondents recommended that it was necessary to pay attention to the size and suitability of the content when designing buttons, icons, and images and indicated that users could easily understand the given elements. These suggestions related to button and icon labels that did not represent the intended content. In addition, the respondents indicated that use of different colors should be considered because the used colors tended to make features less visible:

The button on the meditation player still doesn't understand, that means being pushed or next to the next session. Maybe the icon can be matched like the one that is often used in many applications.

Respondent 4

The image in the mood tracker feature is still too invisible; I suggest that the color can be brightened even more.

Respondent 2

I think the flow on the target is already drawn, but the color is still annoying for me in the activity target feature. At first I saw, the color was very exciting, so I had to press the button several times before I understood.

Respondent 3

The questionnaire data analysis for this iteration focused on the results of the usability testing framework (PSSUQ) and design feedback. The PSSUQ results are shown in Table 5.

The PSSUQ has 4 assessment components: system usefulness, information quality, interface quality, and overall. Lewis [47] asserted that design assessments are the best measurement, with smaller values indicating better quality. Overall, the third iteration results were ”good.“ Broadly speaking, the 4 components had values below 4, the middle value, indicating that the third iteration design was better than the second iteration design regarding ease of use, information presented, and interface appearance. Although the third iteration design had better values than the previous iteration, there were still some improvements needed. Suggestions included paying more attention to the use of color, especially in the activity target feature, so as not to obscure the existing flow, and incorporating effortless design by reducing the number of clicks users must make. Respondents recommended the app’s activity target feature that employs submenu options for each existing target list.

**Table 3:** Demographic characteristics of the respondents who completed online questionnaires in the second iteration (n=108).

Demographic characteristics			Results, n (%)
**Gender**			
	Female	60 (60)	
	Male	48 (48)	
**Age (years)**			
	<17	21 (21)	
	17-23	79 (79)	
**Occupation**			
	High school student	12 (12)	
	College student	75 (75)	
	Psychologist	1 (1)	
	Doctor	1 (1)	
	Others	11 (11)	

**Table 4:** Demographic characteristics of the respondents who completed the questionnaire in the third iteration (n=252).

Demographic characteristics			Results, n (%)
**Gender**			
	Female	161 (63.9)	
	Male	91 (36.1)	
**Age (years)**			
	<17	59 (23.4)	
	17-23	193 (76.6)	
**Occupation**			
	High school student	68 (27)	
	College student	153 (60.7)	
	Others	31 (12.3)	

**Table 5:** Summary of the Post-Study System Usability Questionnaire (PSSUQ) assessment results in the third iteration (more than the middle value of 4 and close to 1 is considered good).

Assessment item	Results
System usefulness	2.341
Information quality	2.597
Interface quality	2.261
Overall	2.416

## Discussion

### Principal Findings

This study designed and developed an app prototype for people with symptoms of depression and tailored it to users’ needs. To create an app, data are collected to identify user needs and user problems. From the responses from the interviewees, there was no difference in feature requirements among the respondents involved; thus, this app’s design could provide support for mental health patients and health workers. Wang et al [48] stated that mental health apps have the potential to improve the monitoring and management of mental health symptoms or disorders. The potential direct benefits of using the mental health app are prevention of higher-acuity illness, higher rate of psychiatrist use, increased competition of services driving lower treatment costs, lower operating costs for psychiatrists, fewer missed appointments, and revenue for app developers [49]. Moreover, Powell et al [49] stated potential indirect benefits of using mental health apps, namely improved physical health, enhanced current and future productivity, and reduced demands on caregivers. In this study, medical personnel also underlined that the use of the mental health app made it easier for them to conduct consultation sessions. This is because the use of the app can generate reports based on the activities recorded by the user in the app.

Moreover, this study found that mHealth features can be classified into main and supporting features. The main features that need to be present for the mHealth app to focus on treating depression are a mood tracker, community, activity targets, and meditation. In the mood tracker feature, information related to the content of thoughts, feelings, and journals needs to be included but is optional. This is because these 3 components can help experts treat patients more accurately. Furthermore, in the community feature, the topics provided should be diverse and cover various problems so that users are more free to find support. The activity target feature was emphasized to provide options in creating activity targets, namely manually and the app’s choice. This is designed so that experts and patients can adjust the target with the therapy or treatment that is being carried out. There are several notes for the feature. The notes given regarding the main feature were that the meditation feature must consider the meditation topic that should exist. This is because meditation sessions can make users spin in their minds, creating the potential for negative thoughts to arise. In addition to the main features, supporting features were suggested to complement the design, namely articles and early detection. The article feature helps provide education to users regarding depression, and the early detection feature provides assistance so that users can screen for their condition. In the article feature, it is better to display several categories of information delivery so that information can be received not only through text but also through images or videos. For early detection features, it is recommended to use the Personal Health Questionnaire (PHQ). This is because the PHQ is already available in Indonesian and widely used by practitioners to screen the initial condition of a person's mental health.

The first discovery involved features and components that need to be considered in designing apps for users who experience depression. We found that many existing mental health apps included mood tracker, activity target, community, and meditation features. However, the features’ requirements for users with depression differ from similar features in the observed health apps. The mood tracker is important to correctly evaluate the user's condition and helps mental health professionals provide more precise patient management. In addition, we found that some debate surrounds the meditation feature because it can leave users trapped in their thoughts, so existing topics must arouse the user's enthusiasm.

We also found that users require concise sentences in the design, which is consistent with the finding by Fanfarelli et al [50] that shorter sentences help to highlight an instruction. Moreover, based on the results of the interviews, the development of this prototype requires integration with health facilities so that it can form a digital ecosystem that can connect with required mental health workers. In addition, a future challenge is the need to develop mental health apps that are secure (specifically could manage patients’ data privacy), simple, and easy to use so that they continue to increase the use of these apps. This finding is also in line with those of Graham et al [12] and Torous et al [51].

### Implications

In contrast with the study by Kenny et al [20] on designing mental health apps in terms of the UX, our study focused on priority features and the support required. The results showed that the main required features were the mood tracker, activity target, community, and meditation and that education articles and early detection were supporting features. In addition to providing information about required features, our study adds to previous research results on the user interface (UI), revealing that the use of color plays an important role in the design; in addition to affecting the intended design use, muted colors should be used in the design (eg, light blue, purple) to reduce or prevent users’ confusion.

In contrast to the study by van Dooren et al [21], which focused on experts’ monitoring of patient use results, our study evaluated the design using both professionals and intended users (adolescents with depression), allowing us to identify the needs and perspectives of both parties. Although it is rich in point of view, it is necessary to confirm this design’s effectiveness for evaluating counseling activities to validate the design results. Our approach also differed from the theoretical approach used by Fanfarelli et al [50], which did not show whether their results were consistent with app users’ expectations. Our approach evaluated both theory and practice to better identify whether users’ goals and expectations were met. Our interview methods also differed from those of Fanfarelli et al [50], which focused on UX using a pretest/posttest design, while our study focused on user needs, UX, and UI, with feedback obtained from 2 flows: the user and user action when using the app. Finally, this study also found that it is important to determine whether the app design can educate users and suit their needs. In accordance with Kenny et al [20], Fanfarelli et al [50], and van Dooren et al [21], it is important to determine user needs and priorities when designing apps to reduce unnecessary information.

### Limitations

Due to the different health management regulations in each country, this study only focused on the Indonesian context. This design can be used as a reference for different country contexts if they have similar mental health treatment for adolescents. Moreover, a limitation of this study is the involvement of only adolescent respondents in urban areas. This research only produced a high-fidelity prototype and must be developed further.

### Conclusions

This study designed the main and supporting features for mHealth apps that focus on managing adolescent depression. Key features include a mood tracker, community, activity target, and meditation, and supporting features that complement the design include education articles and early detection features. The results of this study can be a reference to develop specific mHealth apps to treat and manage adolescent depression in the future. Further research should design and evaluate mHealth apps that can be integrated with other systems, which will allow apps to assist with a broader range of mHealth service needs in addition to depression.

## Appendix

Multimedia Appendix 1 System Usability Scale (SUS) questions.

Multimedia Appendix 2 Post-Study System Usability Questionnaire (PSSUQ).

Multimedia Appendix 3 Final main page design results with all features.

## Abbreviations

DSR: design science research
mHealth: mobile health
PHQ: Personal Health Questionnaire
PSSUQ: Post-Study System Usability Questionnaire
SUS: System Usability Scale
UI: user interface
UX: user experience

## Data Availability

The data sets during generated or analyzed during this study are not publicly available due to the lack of authority to share data.
