# Supplementary material for: Designing High-Fidelity Mobile Health for Depression in Indonesian Adolescents Using Design Science Research: Mixed Method Approaches
Source: JMIR Form Res. 2023 Jul 3;7:e48913. doi: 10.2196/48913 (PMC10365601; doi:10.2196/48913)
Supplement: Multimedia Appendix 1 [file formative_v7i1e48913_app1.docx]

System Usability Scale questions

| **Code** | **Questionnaire Item** |
| --- | --- |
| S1 | I think I **will use this application** often |
| S2 | I think this application is **too complex** (displays many unnecessary things) |
| S3 | I find this application **easy to use** |
| S4 | I need **technical assistance** (tutorial / FAQ) to use this application |
| S5 | I think the **features** provided in this application are **well designed** |
| S6 | I judge too **many inconsistencies** in this application (example: features, images, explanations) |
| S7 | I feel **most people** will quickly **find** **it easy to use** this application |
| S8 | I think this **application flow** is **very complicated** to use |
| S9 | I feel **very confident** using this application |
| S10 | I **need to learn many things** to use this application properly |
