# Supplementary material for: Designing High-Fidelity Mobile Health for Depression in Indonesian Adolescents Using Design Science Research: Mixed Method Approaches
Source: JMIR Form Res. 2023 Jul 3;7:e48913. doi: 10.2196/48913 (PMC10365601; doi:10.2196/48913)
Supplement: Multimedia Appendix 2 [file formative_v7i1e48913_app2.docx]

Post-Study System Usability Questionnaire

| **Dimension** | **Description** | **Code** | **Questionnaire Statement** |
| --- | --- | --- | --- |
| *System Usefulness* | A system that can be easily learned and used, can effectively complete its tasks, and can quickly increase the productivity of its users. | P1 | Overall, I am **satisfied** with the **ease of use** of the application |
|  |  | P2 | I find it **easy to use** this application |
|  |  | P3 | I can **complete** tasks and scenarios **fast** through this application |
|  |  | P4 | I feel **comfortable using** this application |
|  |  | P5 | I find it **easy to learn** how to use this application |
|  |  | P6 | I **will be productive** using this application |
| *Information Quality* | The information provided by the system can be easily understood, can effectively assist in task completion and organization. | P7 | I **got an error message** explaining how to fix it |
|  |  | P8 | Whenever I make a mistake in the application, I **can easily and quickly fix** it |
|  |  | P9 | I feel the **help information** provided by the application is **very clear** |
|  |  | P10 | I find it **easy to find** the **information** needed |
|  |  | P11 | The **information** provided by the app **was effective** in helping me work on assignments and scenarios |
|  |  | P12 | The **information arrangement** provided by the application is **quite clear** |
| *Interface Quality* | The system created provides satisfaction to its users both in appearance and as a whole | P13 | I feel the **interface** is quite **comfortable** |
|  |  | P14 | I like the **interface** in the application |
|  |  | P15 | I feel that **all the functions** and **capabilities** provided by the application have **met my expectations** |
|  |  | P16 | Overall, I am **satisfied** with this application |
