# Supplementary material for: Designing High-Fidelity Mobile Health for Depression in Indonesian Adolescents Using Design Science Research: Mixed Method Approaches
Source: JMIR Form Res. 2023 Jul 3;7:e48913. doi: 10.2196/48913 (PMC10365601; doi:10.2196/48913)
Supplement: Multimedia Appendix 3 [file formative_v7i1e48913_app3.docx]

Final main page design results with all features

| 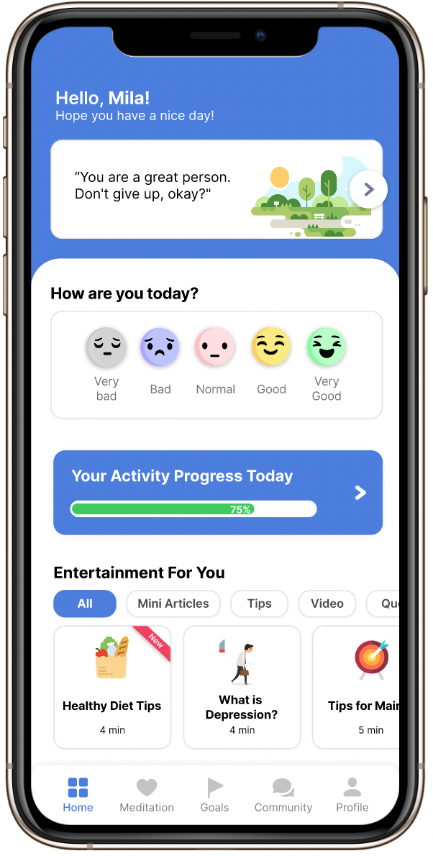  Home Page | 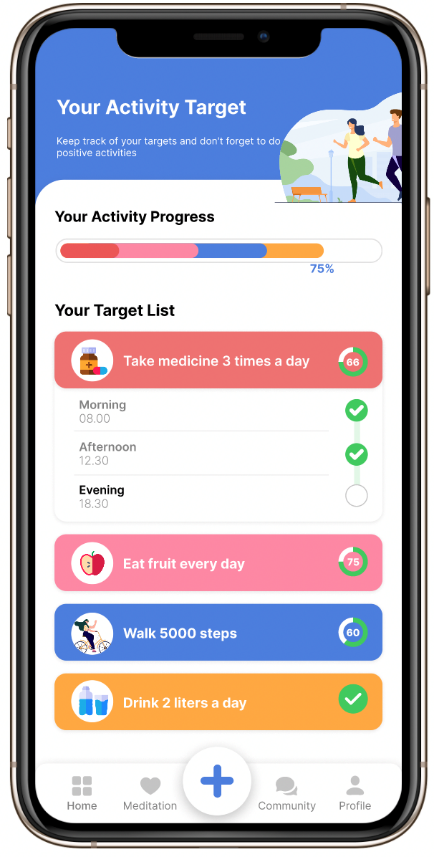  Activity Target Main Page | 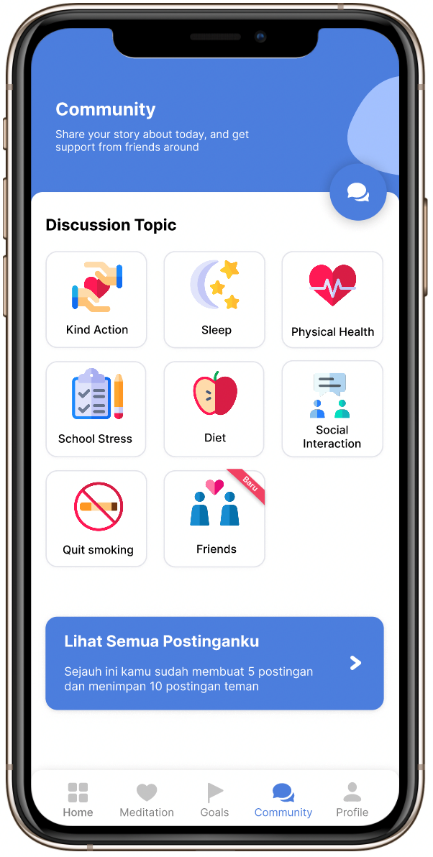  Community Main Page |
| --- | --- | --- |
| 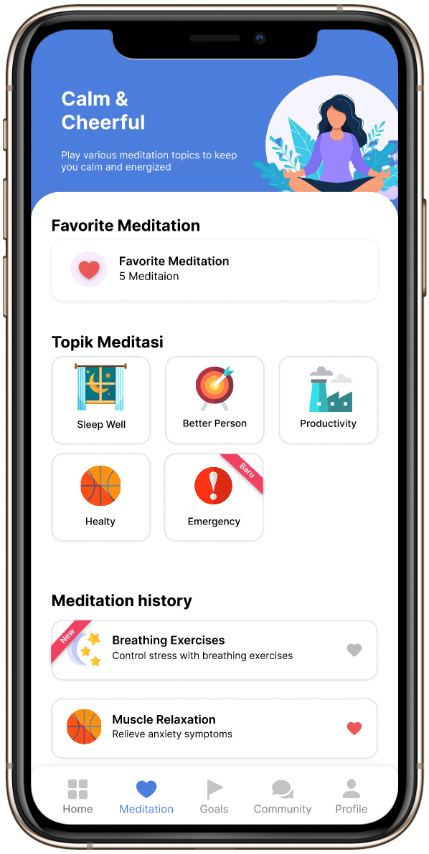  Meditation Main Page | 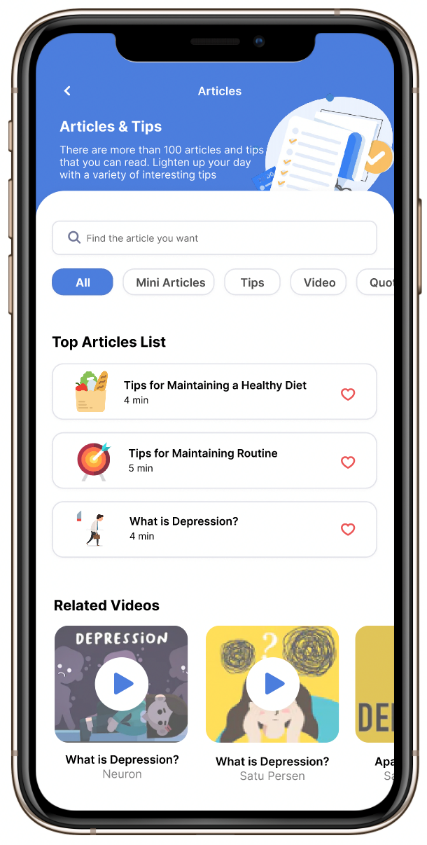  Articles Main Page |  |
